# Recombinant COVID-19 vaccine based on recombinant RBD/Nucleoprotein and saponin adjuvant induces long-lasting neutralizing antibodies and cellular immunity

**DOI:** 10.3389/fimmu.2022.974364

**Published:** 2022-09-08

**Authors:** Amir Ghaemi, Parisa Roshani Asl, Hedieh Zargaran, Delaram Ahmadi, Asim Ali Hashimi, Elahe Abdolalipour, Sahar Bathaeian, Seyed Mohammad Miri

**Affiliations:** ^1^ Department of Influenza and other respiratory viruses, Pasteur Institute of Iran, Tehran, Iran; ^2^ TRS Biotech Company, Tehran, Iran; ^3^ TRS Biotech Company, Auckland, New Zealand

**Keywords:** SARS-CoV-2, RBD, nucleocapsid, vaccine, recombinant protein, neutralization antibody, CD4+ and CD8+

## Abstract

SARS-CoV-2 has caused a global pandemic, infecting millions of people. An effective preventive vaccine against this virus is urgently needed. Here, we designed and developed a novel formulated recombinant receptor-binding domain (RBD) nucleocapsid (N) recombinant vaccine candidates. The RBD and N were separately expressed in *E. coli* and purified using column chromatography. The female Balb/c mice were immunized subcutaneously with the combination of purified RBD and N alone or formulated with saponin adjuvant in a two-week interval in three doses. Neutralization antibody (Nabs) titers against the SARS-CoV-2 were detected by a Surrogate Virus Neutralization (sVNT) Test. Also, total IgG and IgG1, and IgG2a isotypes and the balance of cytokines in the spleen (IFN-γ, Granzyme B, IL-4, and IL-12) were measured by ELISA. The percentages of CD4+ and CD8+ T cells were quantified by flow cytometry. The lymphoproliferative activity of restimulated spleen cells was also determined. The findings showed that the combination of RBD and N proteins formulated with saponin significantly promoted specific total IgG and neutralization antibodies, elicited robust specific lymphoproliferative and T cell response responses. Moreover, marked increase in CD4+ and CD8+ T cells were observed in the adjuvanted RBD and N vaccine group compared with other groups. The results suggest that the formulations are able to elicit a specific long-lasting mixed Th1/Th2 balanced immune response. Our data indicate the significance of the saponin-adjuvanted RBD/N vaccine in the design of SARS-CoV-2 vaccines and provide a rationale for the development of a protective long-lasting and strong vaccine.

## Introduction

The current disastrous pandemic of COVID-19, caused by the severe acute respiratory syndrome coronavirus 2 (SARS-CoV-2), has led to millions of infections and deaths worldwide (1). SARS-CoV-2 is an enveloped positive sense RNA virus and member of the betacoronavirus genus. More than any other pandemic has highlighted the importance of vaccines and vaccination in human well-being. The time restrictions for producing efficient vaccines, especially when no competent drugs are available for treatment, opened new challenges to vaccine developers to rattle through developing potent COVID-19 vaccines with limited prescience and resources. Another challenge for vaccinologists is virus mutations over time, which give rise to new variants (2). Therefore, there is a need to develop vaccines with the potential to protect against all the variants of SARS-CoV-2 (3).

More than 200 COVID-19 vaccines have been announced by now. Some have been approved for emergency use and administered in a limited or wide range of population, while the others are under preclinical and clinical investigations (4). Most of the approved vaccines are categorized as inactivated, adenovirus-delivered, mRNA-based, and recombinant proteins vaccines (5).

The chief SARS-CoV-2 structural proteins are spike (S), nucleocapsid (N), membrane (M), and envelope (E). The trimeric aggregate of the spike protein of SARS-CoV-2 plays a key role in the virus entry into the host cell by employing the host angiotensin-converting enzyme 2 (hACE2) present at the surface of many human cells. Therefore, research groups have focused on studying the S protein or its fragments as the antigen for vaccine development. This type of vaccines is categorized among the most immunogenic in combination with adjuvants (6).

It has been demonstrated that the spike protein binds to the hACE2 receptor through the receptor-binding domain (RBD), which is necessary for virus entry. Moreover, the C-terminal S2 subunit of spike protein, using heptad repeat 1 (HR1) and heptad repeat 2 (HR2), mediates the connection between the viral envelope and cellular membrane (4). In this regard, it has been observed that following infection with SARS-CoV-2 or COVID-19 vaccination, the most important neutralizing antibodies are against RBD, suggesting this region as a well-suited candidate for vaccine design and thereby directly blocking the virus entry into the host cell (7, 8). This observation is explained by the fact that the RBD fragment is less glycosylated than other parts of spike protein and more accessible to antigens (9). Accordingly, some studies have incorporated RBD, whether as a single monomer antigen or in dimeric or trimeric form with or without other antigen(s) and adjuvants, to construct recombinant COVID-19 vaccines (10–14). A recombinant vaccine comprised of residues 319 to 545 of RBD adjuvanted with aluminium hydroxide gel potently evoked antiviral antibody in animal and non-human primates models. Some important benefits of this candidate vaccine are eliciting strong functional antibodies 1 or 2 weeks after immunization, blockade of RBD-ACE2 binding, protection against SARS-CoV-2 live and pseudovirus, involvement of lymphocytes with the ability to stimulate IFN-γ and IL-4 production, rise in the level of serological neutralizing antibodies specific of RBD, and recruitment of both CD4 and CD8 memory T cells without any detectable histopathological alterations (15).

However, despite all advantages attributed to RBD-based vaccines, its small molecular size may result in poor immunogenicity. In this respect, incorporation of more than one antigen, whether in the form of multimers or distinct antigens administered in fused or separate forms, has shown a synergistic role by eliciting stronger antiviral antibody responses. Besides, using immunopotentiator adjuvants to increase the titer and durability of antibody responses is of great importance and has been widely reported. One such example is a trimeric RBD vaccine adjuvanted with toll-like receptor 7/8 (TLR-7/8) agonist adsorbed to alum (alum-3M-052). This vaccine showed potency in inducing anti-SARS-CoV-2 antibody responses against the live virus and in protecting the mice against virus challenge, highly more than that of non-adjuvanted monomer antigen (16). Also, a comparative study between the adjuvant capacity of three lipophilic adjuvants in the vaccines comprised of SARS-CoV-2 spike protein or its fragments reported monophosphoryl lipid A as a potent adjuvant in eliciting high levels of humoral and cellular immune responses (17).

In addition to spike protein, some studies have employed nucleocapsid protein as an immunogen for vaccine design. Moreover, it has been very recently reported that co-immunization with spike and nucleocapsid protein of SARS-CoV-2 provides protection both in proximal and distal organs. This protection has not been observed in their individual administration, introducing N protein as a suitable antigen candidate for COVID-19 vaccine development (18). The new variants of the virus may find ways to escape neutralizing antibodies. Accordingly, the capability of vaccines to induce sufficient T cell-mediated cellular and humoral immune responses is of high importance. In this regard, previous studies have introduced some of the SARS-CoV-2 N and S protein fragments as an inducer of potent T cell responses, highlighting their valuable capacity for vaccine production (19). As coronavirus’ most abundant structural protein, Nucleocapsid protein, as the most abundant structural protein of coronavirus, harnesses the cross-reactive T cell epitopes, justifying its impact on augmentation of T cell responses. While this protein has a slight effect against initial virus infection of the respiratory system, its role in controlling the further progress of infection to distal parts by eliciting N-specific T cells and thereby killing infected cells seems pivotal (18).

Moreover, many types of adjuvants can be used to enhance the immunogenicity of vaccines. Among these adjuvants, saponins (as natural glycosides of steroid or triterpene) have the potential to elicit the immune system of mammals, especially by enhancing both Th1 immune response and the production of cytotoxic T-lymphocytes (CTLs) against exogenous antigens. Therefore, they can be considered among ideal vaccine adjuvants (20).

In the present study, we evaluate the safety, immunogenicity, and protective efficacy of our developed RBD/Nucleoprotein vaccine adjuvanted with saponin. This candidate vaccine harnesses specific neutralizing antibodies and T cell responses provoked by both RBD and N proteins. Therefore, it has the synergistic effect of N protein in designing and constructing recombinant vaccines based on spike protein. Mouse studies demonstrated that the adjuvanted RBD/Nucleoprotein vaccine elicits a higher neutralizing antibody response against the SARS-CoV-2 virus than other individual or combinatorial formulations. These data support the utility of adjuvanted RBD/Nucleoprotein as a potential vaccine candidate against SARS-CoV-2 infection.

## Materials and methods

### Expression of N and RBD proteins in *E. coli*

The genes codon-optimized encoding SARS-CoV-2 S protein RBD and Nucleoprotein were artificially synthesized (Biomatik, Canada) and sub-cloned into pET22b (+) and pET28a vectors using restriction endonucleases. The design, expression, and purification of the RBD construct was conducted according to the previous publication (21).

The recombinant protein was expressed by transforming the recombinant plasmid pET22b-RBD and pET28a-N into *E. coli* strain BL21(DE3) using a heat shock method and cultured in an LB agar plate containing 100 μg/mL ampicillin and 50μg/ml kanamycin. Then, the selected colonies harboring recombinant plasmids were grown in LB broth at 37°C with shaking at 180 rpm. The expression of proteins was induced by 1 mM isopropyl-β-d-thiogalactoside (IPTG, Sigma, St. Louis, MO) at optical density (OD)= 0.6 - 0.8 and 18 h incubation at 37°C while shaking at 190 rpm. The induced cells were harvested by centrifugation at 9000 rpm for 20 min at 4°C. Protein expression was determined by sodium dodecyl sulfate-polyacrylamide gel electrophoresis (SDS-PAGE) using the method of Laemmli. Also, the molecular weights of RBD and N proteins were estimated with a standard marker. Finally, the gels were stained with Coomassie blue R-250.

### Extraction and purification of the recombinant proteins

**Figure 1:**
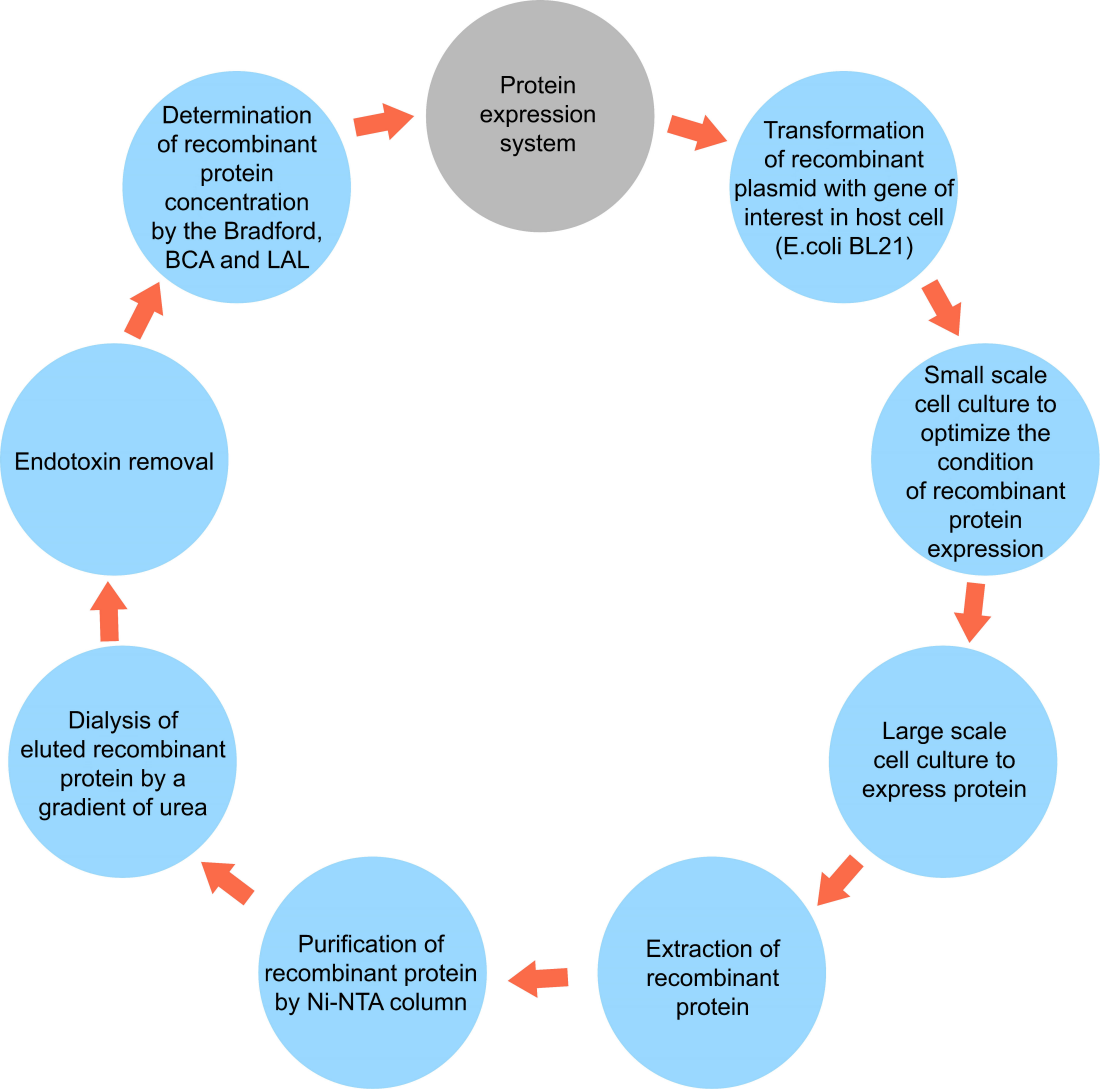
A simplified schematic diagram of protein expression system including extraction and purification.

After harvesting the bacteria by centrifugation, for cell lysis and extraction of the recombinant proteins, the bacterial pellets were lysed in the lysis-equilibration-wash (LEW) (50mM NaH_2_PO_4_, 300mM NaCl, 8M Urea, pH = 8) buffer. Next, they were incubated at 4°C for 30 min and sonicated. This process was repeated two more times to extract all the proteins. The final supernatant containing the desired protein was collected for purification with a Ni-NTA column. For this purpose, the extracted lysate was loaded on the Ni-NTA column (Sigma). Bound polyhistidine-tagged proteins were eluted by a gradient of imidazole with an Elution Buffer containing 50mM NaH_2_PO_4_, 300mM NaCl, 8M urea, and 2.5-250 mM imidazole, pH 8. Afterward, they were refolded by dialysis from 6 M, 4 M, and 2 M to 0 M urea in buffer (20 mM NaH_2_PO_4_, 300 mM NaCl, 2 mM β-mercaptoethanol, 0.4% arginine, 10% glycerol, pH 7.5). SDS-PAGE was performed to analyze the fractions collected from different steps of purification. Finally, the purified protein was dialyzed in Phosphate Buffer Saline (pH 8.5). Also, low-weight substances such as urea and salt were removed by a dialysis membrane bag, as demonstrated in **Figure 1**. Finally, the amount of purified proteins was measured by colorimetric bicinchoninic acid assay (BCA) (Pierce™ BCA Protein Assay Kit, ThermoFisher Scientific) according to the manufacturer’s protocol and stored at −80°C until use.

### Endotoxin removal and quantitation

Endotoxins were removed from the purified protein by porous cellulose bead surface-modified with covalently attached poly(ϵ-lysine) chains (Pierce High Capacity Endotoxin Removal Spin Column, 1 ml, Thermo Fisher Scientific, Inc., USA) with high affinity for endotoxins. After endotoxin binding to the cellulose beads, the beads were equilibrated with an endotoxin-free buffer containing 10-50 mM Tris-HCl buffer containing 0.1-0.2 M NaCl (pH 7) according to the manufacturer’s instructions.

According to the manufacturer’s directions, endotoxin levels in the purified protein were calculated using Pierce™ LAL Chromogenic Endotoxin Quantitation Kit (Thermo Fisher Scientific, Waltham, MA, USA).

Endotoxin concentration in the sample was determined in duplicate by using the standard curve provided by the kit, and results were expressed as EU/ml.

### Western blot analysis for the recombinant proteins

Recombinant protein samples were electrophoresed on 12% polyacrylamide gel and then transferred to a nitrocellulose membrane (Sartorius, Germany). The expected N and RBD proteins were detected using monoclonal antibody SARS-CoV-2 Nucleocapsid Protein (HL344) and SARS-CoV-2 Spike Protein (RBD) (E2T6M) as primary antibody and convalescent serum of COVID-19 patients, respectively. Moreover, an anti-mouse IgG HRP-linked antibody was applied as the secondary antibody. In addition, regarding the presence of a polyhistidine sequence in the recombinant proteins, the proteins were monitored using an Anti-His tag antibody (His-Tag (27E8) Mouse mAb #2366). Finally, the proteins band were visualized using Diabimabenzidine (DAB, Sigma, UK) substrate. Cell lysate before induction was used as the negative control.

### Balb/c mice immunization

A total of 70 female mature Balb/c mice were housed in the animal facility at the Pasteur Institute of Iran, Tehran, Iran, and randomly assigned into seven groups. Two groups were injected with 15 μg RBD alone or supplemented with saponin adjuvant. Two groups received N protein with or without saponin adjuvant. The other two groups were injected with the mixture of RBD and N with or without saponin adjuvant.

**Figure 2:**
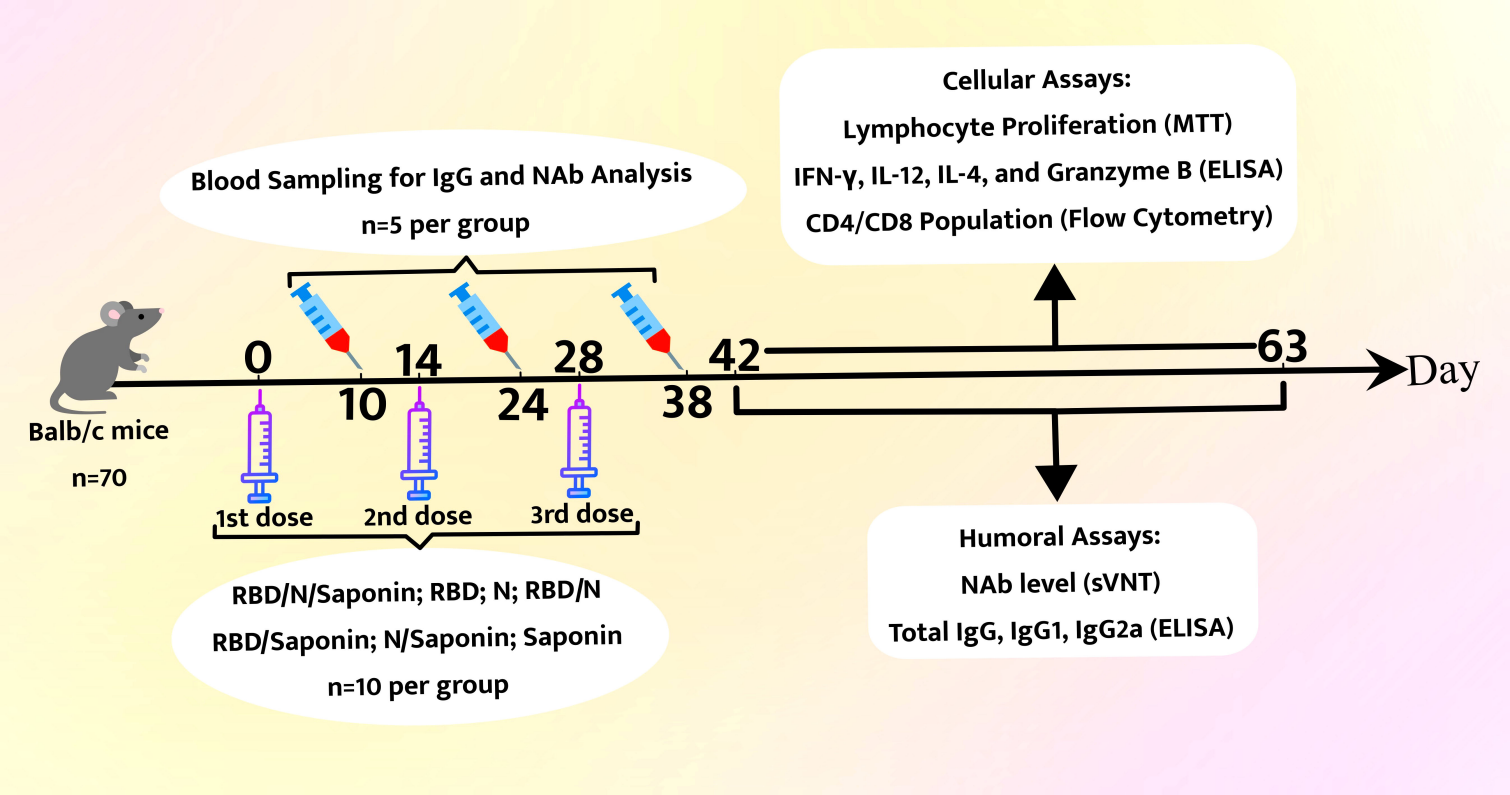
Schematic overview of Mouse immunization and analysis schedule.

As a control, mice were given 15 μg saponin alone. The saponin was supplied by *In vivo*Gen and was dissolved in Milli-Q water. Mice were injected subcutaneously at two-week intervals in three doses. The recombinant protein dose used in all groups was equal to 15 μg per injection in a total volume of 100 μl. All these procedures were performed based on the protocols approved by the Ethics Committee. Different aspects of immunity (i.e., lymphocyte proliferation (MTT), cytokine levels (ELISA), and CD4/CD8 population (flow cytometry) were studied two and nine weeks after the third administration. Serum samples were collected 10 days post each vaccination to detect IgG and neutralize antibody responses. Results are representative of three independent experiments (three mice per group). Statistical analysis was done using Student’s t-test. The final data represent the mean ± standard deviation (S.D.) of three measurements. The schematic overview of all experimental procedures is depicted in **Figure 2**.

### Specific IgG responses

Blood samples were collected from individual mice of each group (n=5) *via* orbital sinus bleeding two and nine weeks post-last vaccination. Next, serum levels of SARS-CoV-2 anti-RBD and anti-N total IgG, IgG1, and IgG2a were assayed by enzyme-linked immunosorbent assay (ELISA) for total specific anti-RBD and anti-N IgG using ELISA technique, as previously described (22, 23). Briefly, ELISA plates were coated with 100 ng/well RBD synthetic peptide (Biomatik) and N synthetic peptide (Biomatik) (in coating buffer) with a final concentration of 10^-4^ mg/ml. Afterward, they were sequentially incubated with diluted serum samples (1:1000 dilutions) and horse radish peroxidase (HRP)-conjugated goat anti-mouse IgG, IgG1, or IgG2a (Sigma-Aldrich) for 2 h at room temperature. Next, HRP substrate was added to the mix to develop the colorimetric assay. After stopping the reaction with 2 M H_2_SO_4_, the values were determined by measuring the optical density (OD) of ELISA plate wells at 450 nm. The endpoint ELISA titers were determined as the highest serum dilution generating a stronger signal (at least 3 times) compared to OD values of samples from control mice at equivalent dilutions. Serum dilutions of 10^2^ to 10^8^ were used to determine IgG titers. Moreover, ELISA endpoint titers were determined as the highest reciprocal serum dilution that yielded an absorbance >2-times over more than the background values.

### SARS-CoV-2 surrogate virus neutralization test

The neutralization antibody titers against the SARS-CoV-2 were detected by a Surrogate Virus Neutralization (sVNT) Test. The competitive ELISA assay measures the relative amount of neutralizing antibodies against a calibrator using a standard curve (**Figure 5**). The results are then displayed as titer according to the manufacturer’s instructions.

An ELISA plate (Nunc) was pre-coated with 100 ng human ACE2 protein (GenScript) in carbonate-bicarbonate coating buffer (pH 9.6) overnight at 4°C, followed by blocking. For the direct binding assay, HRP-conjugated SARS-CoV-2 RBD (GenScript) was added to the hACE2-coated plate at different concentrations for 1 h at room temperature. A colorimetric signal was developed on the enzymatic reaction of HRP with a chromogenic substrate, 3,3’,5,5’-tetramethylbenzidine (TMB) (Sigma). After stopping the reaction with 2 M H_2_SO_4_, the values were determined by measuring the optical density (OD) of ELISA plate wells at 450 nm.

For the sVNT assay, 3 ng of HRP-RBD (from either virus) was pre-incubated with test serum for 1 h at 37°C (final volume of 50 μl), followed by addition into an ELISA plate coated with hACE2 for 1 h at room temperature. Unbound HRP-conjugated antigens were removed through five PBST washes. Eventually, the inhibition percentage was determined as follows:


$$Inhibition=(1-\frac{OD\;value\;of\;sample}{OD\;value\;of\;negative\;control})\times 100\%$$

### Lymphocyte proliferation assay

Lymphocyte proliferation of splenocytes was evaluated (three mice/group) one week after the last vaccination. The splenocytes at a concentration of 2 × 10^5^ cells/well were propagated in the 96 well plates containing RPMI-1640 supplemented with 10% FBS, 1% L-glutamine, 1% HEPES, and 0.1% penicillin/streptomycin in the presence of 1μg of synthetic RBD (366-374aa)/N (223-231aa)-specific CTL epitope (Biomatik, Canada) (24–27) or culture media as mock stimulated control.

Lymphocyte proliferation was evaluated using the MTT (3-(4,5-dimethylthiazol-2-yl)-2,5-diphenyltetrazolium bromide) kit (Sigma), based on a colorimetric reaction. After 72 hours of incubation at 37°C and 5% CO_2_, 10 μg/μl of the MTT solution was added to each well. Then, they were incubated under the same conditions for five hours. At the end of incubation, the supernatant was removed, and 100 μl of the solution buffer was added to wells to make purple soluble formazan crystals. The plate was read using an ELISA reader (BIOTEK) at 540 nm, and the OD was recorded to calculate the stimulation index (SI). The SI was calculated by subtracting the relative cell numbers of unstimulated cells (Cu) from the OD of stimulated cells (Cs) divided by relative OD values of unstimulated cells; SI = (Cs _ Cu) / Cu (28).

### Cytokine secretion assay

One week after the last immunization, splenocytes of immunized mice were cultured in 24-well plates for 3 days in phenol red-free RPMI 1640 (Thermo Fisher, USA) supplemented with 10% FBS, 2 mM L-glutamine, 25 mM HEPES, and 0.1% penicillin/streptomycin. Next, the mix was pulsed with 1 μg/ml RBD/N-specific CTL epitope or culture media as mock stimulated control at 37°C in 5% CO_2_. The supernatants were assayed for the presence of IFN-γ, IL-4, and IL-12 using commercially available sandwich-based ELISA kits (R&D systems, San Diego, USA), according to the manufacturer’s instructions.

### Analysis of CD4/CD8 population by flow cytometric

The CD4/CD8 population in the spleens of immunized mice was evaluated by analyzing the freshly prepared splenocytes were analyzed by flow cytometry using the eBioscience Mouse Regulatory T Cell Staining Kit. Briefly, splenocytes (1×10^6^/well) were cultured for 5 h in complete RPMI-1640 alone (negative control) or co-cultured with RBD/N-specific CTL epitope antigens. The antibodies and reagents used for staining were FITC-conjugated anti-CD4, APC-conjugated anti-CD8 and PE -labeled CD3 antibody. The samples were analyzed using a BD FaxCalibur flow cytometry device.

### Analysis of granzyme B activity

The influenza-specific cytolytic activity was investigated by measuring the amount of granzyme B (GrB) protein, as a marker of activated cytotoxic T cells, in the supernatants of RBD/N-stimulated mononuclear cells from spleens of three mice in each group. The measurements were performed two and nine weeks after the last administration, according to the instructions from the commercially available granzyme B sandwich-based ELISA kit (R&D, USA). Samples and standards were evaluated at an optical density of 450 nm. All tests were carried out in triplicate for each mouse (22).

### Statistical analysis

Data were expressed as means ± SD. Results between the different groups were compared using the one-way ANOVA test. The statistical significance level was set at P-value ≤ 0.05. In this study, the statistical analyses were performed using the statistical software SPSS ver. 16.0 (SPSS Inc., Chicago, IL, USA) was used for statistical analysis. Correlation analysis was performed by log transforming of the endpoint ELISA or neutralization titers, followed by linear regression analysis.

## Results

### Expression and confirmation of recombinant N and RBD proteins

**Figure 3:**
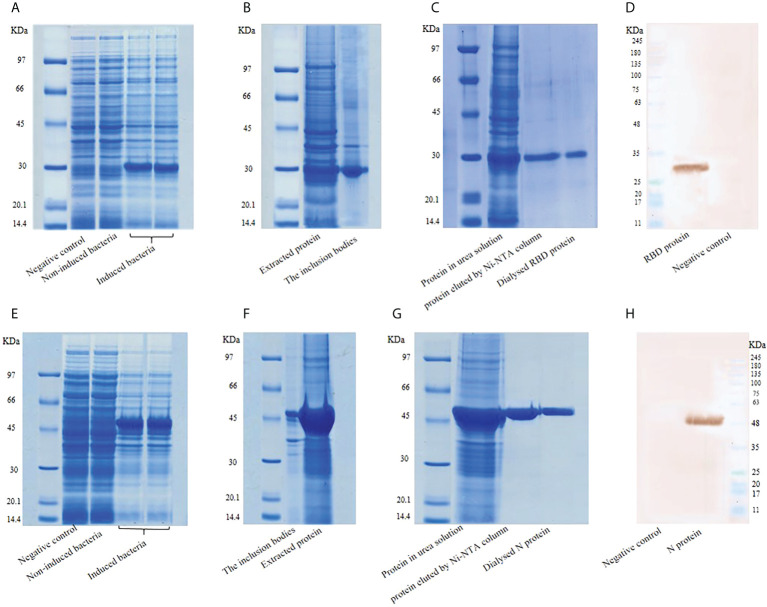
**(A)** Expression of RBD protein, **(B)** Extraction of RBD protein, **(C)** Purification and dialysis of RBD, **(D)** Western blot analysis of RBD. **(E)** Expression of N protein, **(F)** Extraction of N protein, **(G)** Purification of N protein, **(H)** Western blot analysis of N protein.

Recombinant pET22b-RBD was transferred into *E. coli* host strain BL21 (DE3). The cells were harvested at 3.5 hours after 0.5 mM IPTG induction and disrupted by lysis buffer. Recombinant pET28a-N transferred into *E. coli* host strain BL21 (DE3) samples were collected 18 hours after 1mM IPTG induction. The bacterial pellets were disrupted by lysis buffer to obtain the cell-free extract for SDS-PAGE analysis and protein bands were visualized on 12% polyacrylamide gel. As shown in **Figure 3A**, a major electrophoresis band corresponding to ∼29 kDa appeared at the culture with IPTG induction (Lane 4 and 5). This band was absent in the culture without IPTG induction (Lane 3). Thus, RBD was expressed in *E. coli* upon induction with IPTG. Expression analysis of N protein by SDS page demonstrated an approximately ∼46 KDa band as expected (**Figure 3E**). Recombinant proteins expressed in *E. coli* were extracted according to the procedure described in Section 2.2 (**Figure 3B** and **Figure 3F**). Moreover, the recombinant proteins in the cell lysate supernatants were purified with Ni-NTA column chromatography and electrophoresed on 12% SDS-PAGE. The results of SDS-PAGE showed the high specificity of the purification of RBD and N proteins using this system. The purified proteins were finally dialyzed in Phosphate Buffer Saline by a gradient of urea (**Figure 3C** and **Figure 3G**). The concentration of RBD and N proteins determined by Bradford and BCA methods were 0.8 mg/mL and 0.5 mg/ml, respectively. The expressed recombinant proteins were confirmed by western blot using anti-RBD (E2T6M) (**Figure 3D**) and anti-N (HL344) (**Figure 3H**) antibodies. Probing with convalescent serum of COVID-19 patients also showed bands at the same size for the recombinant proteins (data not shown).

These results suggest that the recombinant proteins maintain intact spatial conformation and authentic antigenicity.

### Endotoxin removal and quantitation

After being concentrated with protein concentrators, endotoxin was removed and its content was quantified by P Pierce™ LAL Chromogenic Endotoxin Quantitation Kit. Endotoxin quantitation showed that the endotoxin content in the purified protein was at the normal level (<0.1 EU/ml). The results indicated that most of the impurities had been removed during the purification process.

### Specific IgG responses

**Figure 4:**
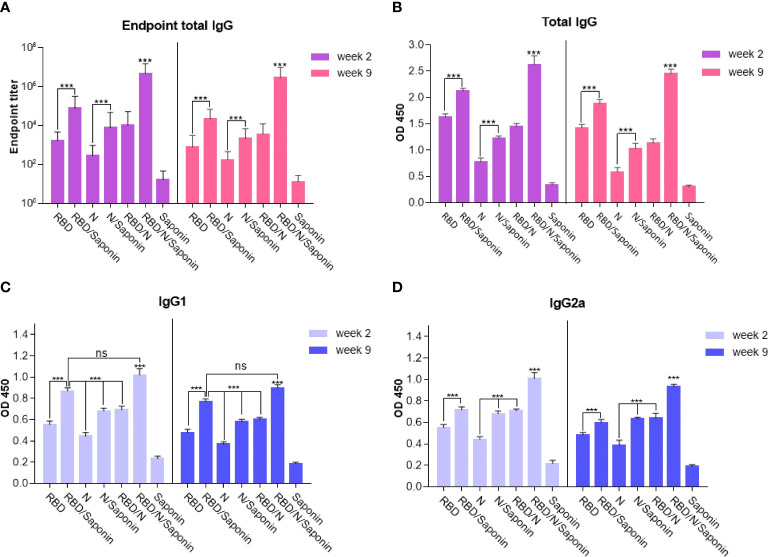
IgG titers of mice immunized with different combinations of RBD and nucleocapsid proteins were determined by ELISA from sera collected on the two and nine weeks after last immunization. Data are expressed as means ± SD of three mice per group and difference among individual groups is determined by One way ANOVA and shown as ***for P < 0.001 and ns for a nonstatistical difference. Compared to *different formulations or control groups*, saponin-adjuvanted RBD/N vaccine significantly induced the highest level of endpoint total IgG and IgG2a isotype.

Humoral responses induced by the recombinant candidate vaccine were evaluated by immunizing the BALB/c mice with different combinations of RBD and nucleocapsid proteins and saponin as the adjuvant three times at two-week intervals. Blood samples were collected two and nine weeks after the last immunization and analyzed for total IgG antibodies specific to RBD and N proteins. Moreover, to determine the Th1/Th2 polarization, the efficacy of each formulation in inducing the IgG subclasses response in mice was evaluated. The levels of IgG1 and IgG2a, as representatives of Th2 and Th1 immunity, respectively, were measured at both weeks. As shown in **Figure 4**, compared to the saponin control, mice immunized with either RBD, N, RBD/N, RBD/saponin, N/saponin, or RBD/N/saponin induced higher levels of total specific IgG, IgG1, and IgG2a both 2 and 9 weeks post-last immunization. In all groups and at both weeks, RBD/N/saponin generated the highest level of specific total IgG compared to all other groups (P<0.001). Concerning other groups, RBD/saponin induced higher (p<0.001) levels of antibody compared to the remaining groups, and no significant difference was observed within other non-control groups at both measurement weeks (**Figure 4B**). However, no significant difference in specific total IgG was observed among RBD, N/saponin, and RBD/N groups.

For IgG1 and IgG2a titer, the following results were observed for both weeks 2 and 9 after the last immunization. Blood analysis demonstrated that while RBD/N/saponin and RBD/saponin groups could elicit comparable levels of antibodies, both induced significantly more IgG1 levels (p<0.001) compared to all other groups (**Figure 4C**). Moreover, a significantly higher level of IgG2a was observed in RBD/N/saponin group compared to all other study and control groups (p<0.001). Combining N protein with either RBD or saponin resulted in a significantly higher level of IgG2a than its individual administration (p<0.001). Furthermore, saponin-supplemented RBD elicited a higher (p<0.001) level of IgG2a compared to RBD alone (**Figure 4D**).

Furthermore, as evident in **Figure 4A**, the endpoint titer of the N/RBD/saponin group was ~10^7^ in this study at week 2 of post-last immunization. More importantly, no significant decrease in endpoint total IgG level was observed at week 9. The total endpoint IgG trend among other groups remained constant from week 2 to 9. The RBD/saponin compared to RBD, and N/saponin compared to N group showed a significantly higher endpoint total IgG (p<0.001). However, no significant difference in endpoint titer was observed among RBD/saponin, N/saponin, and RBD/N groups.

Together, the IgG response results of the saponin-adjuvanted RBD/N vaccine suggest that the adjuvanted formulation of our recombinant protein-based vaccine could effectively induce long-lasting virus-specific IgG against SARS-CoV-2 in vaccinated mice.

### RBD/N/saponin induced noticeable titer of virus-neutralizing antibodies against SARS-CoV-2

**Figure 5:**
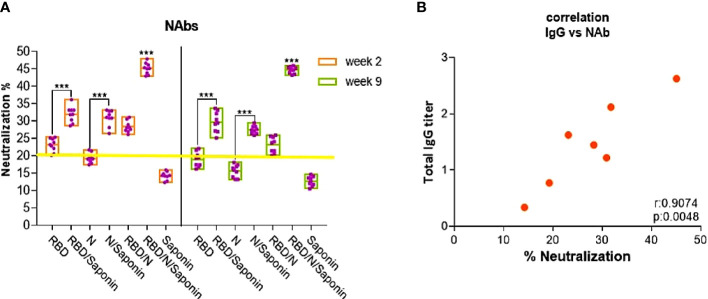
Mean percentages of SARS-CoV-2 neutralizing inhibition. The yellow line represents the sVNT cut-off. PI results < 20% are reported as “negative”, and PI = 20% are reported as “positive” **(A)**. Comparison of specific IgG endpoint titers with SARS-CoV-2-specific neutralizing inhibition. **(B)**. Correlation analysis was performed by log transformation of the endpoint ELISA or NAbs titers followed by linear regression analysis *** for P < 0.001.

In the sVNT, anti-SARS-CoV-2 neutralizing antibodies block HRP-conjugated RBD protein from binding to the hACE2 protein coated on an ELISA plate. The sVNT results indicated significantly higher sVNT inhibition in the mice vaccinated with RBD/N/saponin (45.7 ± 1.6) compared to all control and study groups, suggesting its potent neutralizing effect. The results also showed that both saponin-adjuvanted RBD and N proteins elicited considerably higher levels of sVNT inhibition than their analogous individual forms (p<0.001) (**Figure 5**). Although RBD and N groups were reported as positive (PI ≥ 20%) in sVNT test, their score was highly near the assessment threshold, especially for the N group. Altogether, the sVNT result demonstrates the ability of RBD/N/saponin in eliciting neutralizing antibodies against SARS-CoV-2, emphasizing the enhancement effect of both N protein and saponin adjuvant on RBD in providing humoral immunity after vaccination.

In addition, a moderate decrease was observed in NAbs level at week 9 post-last immunization compared to the one measured at week 2 in all study groups except RBD/N/saponin. However, the results showed no noticeable change in NAbs level for the RBD/N/saponin group, highlighting the efficiency of this combinatorial formula in providing sufficient persistency and protective durability as a potent vaccine candidate.

Correlation analysis using a linear regression model was performed in GraphPad Prism, and Pearson’s correlation coefficient was calculated to understand the relationship between viral neutralization response and specific total IgG antibody titers. The mean titer of SARS-CoV-2 anti-RBD and anti-N total IgG for 3 mice per group was calculated, and the results were analyzed against the mean percentage of inhibition (PI), as representative of neutralization capacity. As shown in **Figure 5B**, there is a strong positive correlation between neutralization (PI) and total IgG titer with Spearman rho=0.758 (p< 0.005). These results suggest that the subset of antibodies has high potency in neutralizing the virus and protecting against upcoming infection and disease. Moreover, it can be inferred that the chief portion of anti-SARS-CoV-2 neutralization antibodies is against RBD and N proteins.

### Saponin-adjuvanted RBD/N candidate vaccine highly enhanced the stimulation of T lymphocyte proliferation

**Figure 6:**
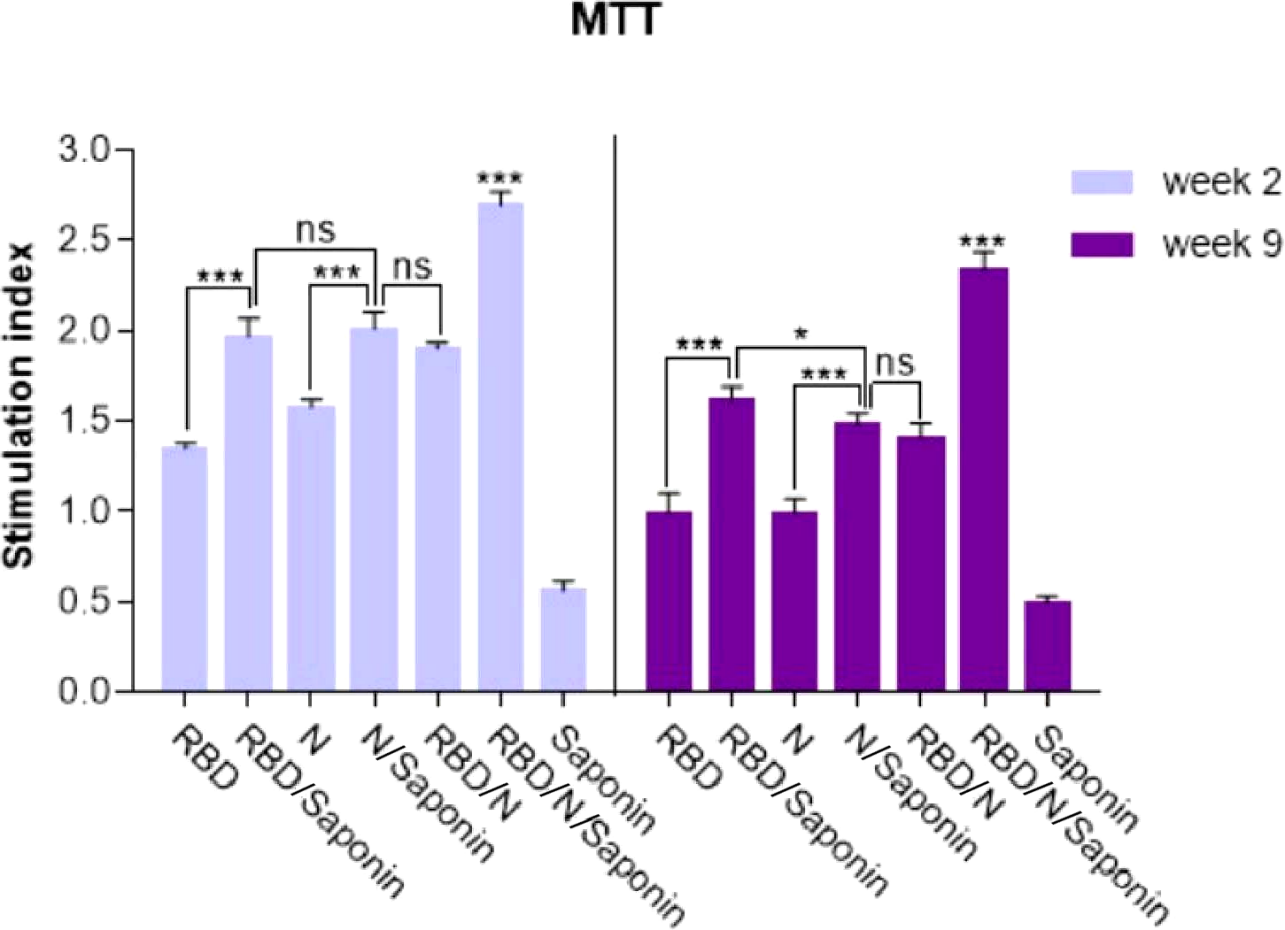
Proliferation of immunized BALB/c mice splenocytes after *in vitro* re-stimulation with synthetic RBD/N specific epitope. Splenocytes from mice were harvested two and nine weeks after the last immunization and lymphocyte proliferation was evaluated by the MTT assay. Results represent the mean ± SD of 3 mice per groups. *** Indicates statistically significant differences between the RBD/N plus saponin adjuvant compared with other groups as determined by one-way ANOVA (P < 0.001). * for P <0.05, and ns for a nonstatistical difference.

The ability of recombinant RBD/N to induce SARS-CoV-2-specific cellular immune responses was evaluated by analyzing the splenocytes of vaccinated animals for antigen-specific lymphoproliferative response by LPA assay. Spleens of three mice in each group were collected 2 and 9 weeks following the last immunization, and the impact of spleen lymphocyte proliferation was analyzed by MTT assay. The results revealed that the stimulating effect of the RBD/N/saponin group was significantly more than that of all other groups (p<0.001) at both weeks. Moreover, higher stimulation index was observed in RBD/saponin group compared to RBD one (p<0.001) and in the N/saponin group compared to the N one (p<0.01) at both weeks (**Figure 6**). Despite a non-significant difference between RBD/saponin and N/saponin at week 2, a higher SI was observed in RBD/saponin group compared to N/saponin group (p<0.05). Except for the control saponin group, comparing the results of those two weeks for each group indicated a decline in SI from week 2 to week 9. However, the decrease in RBD or RBD/N/saponin groups was less than that of the other groups (**Figure 6**).

### Saponin-adjuvanted RBD/N candidate vaccine significantly stimulates the CD4+ and CD8+ induction

To obtain more detailed information on the quality of the observed T cell responses, we further characterized the responsive T cell populations and changes of CD4+ and CD8+ T lymphocytes by flow cytometry.

**Figure 7:**
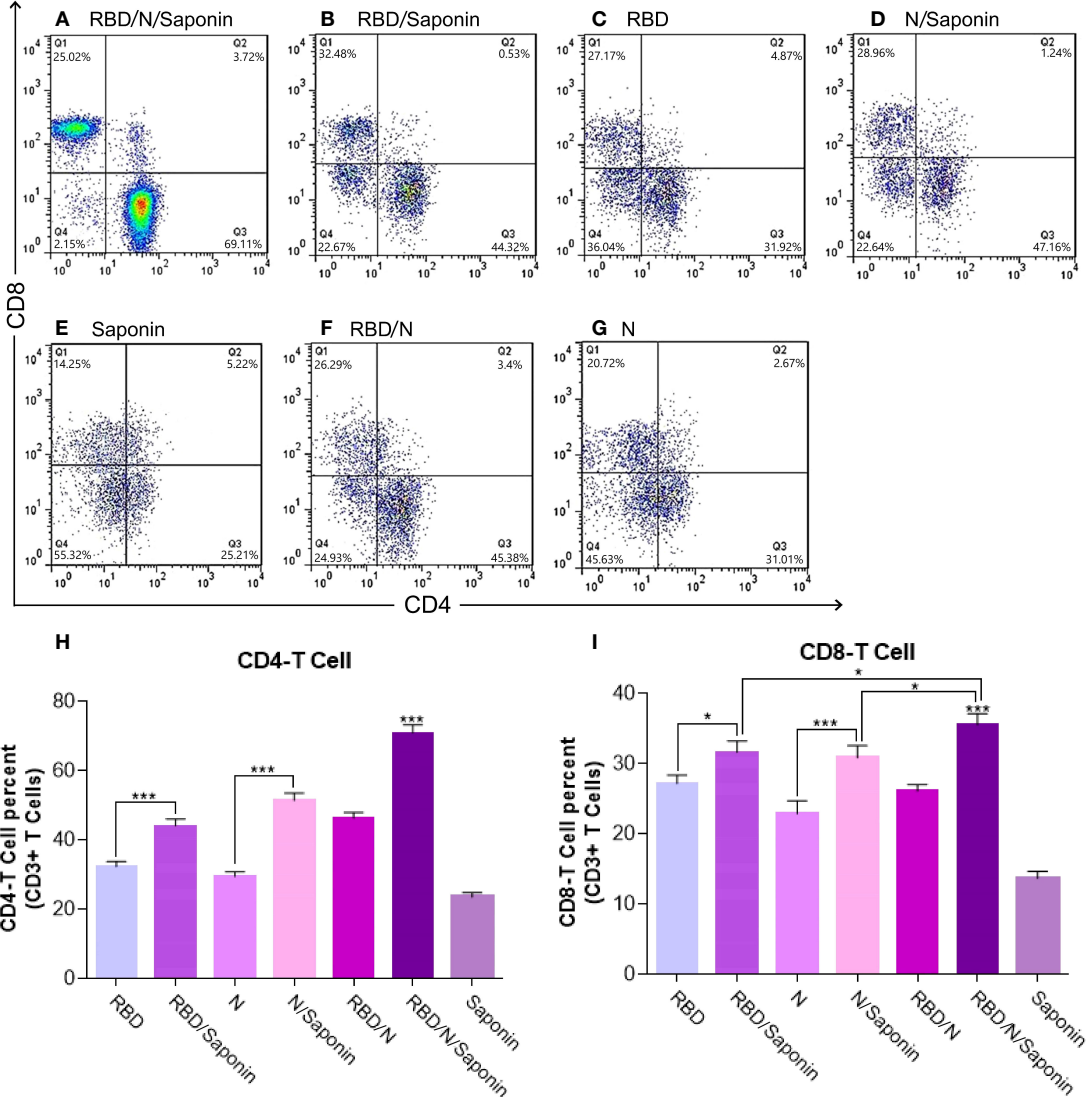
Flow cytometry analysis of levels of CD3 + CD4+ and CD3 + CD8+ T cells in immunized mice. Mouse spleen mononuclear cells were isolated by density gradient centrifugation on Ficoll-Paque. Three different colors were used for CD3+, CD4+ and CD8+ membrane markers. Gated CD3 positive events were analyzed for CD4 and CD8 production. The graph of flow cytometry represents data of five experiments with similar results. The data represent the means ± standard deviation (SD). Statistical analysis between the four groups were analyzed by one-way ANOVA (*p < 0.05, ***p < 0.001).

According to **Figure 7**, immunization with the RBD/N/saponin induced more CD4+ and CD8+ T lymphocytes in the spleen compared to other groups (p<0.001). The flow cytometry results also revealed that compared to the RBD and N groups, the RBD/saponin and N/saponin groups showed higher levels of lymphocytes, respectively.

### RBD/N/saponin induced higher levels of IFN- γ, IL-12, and IL-4

**Figure 8:**
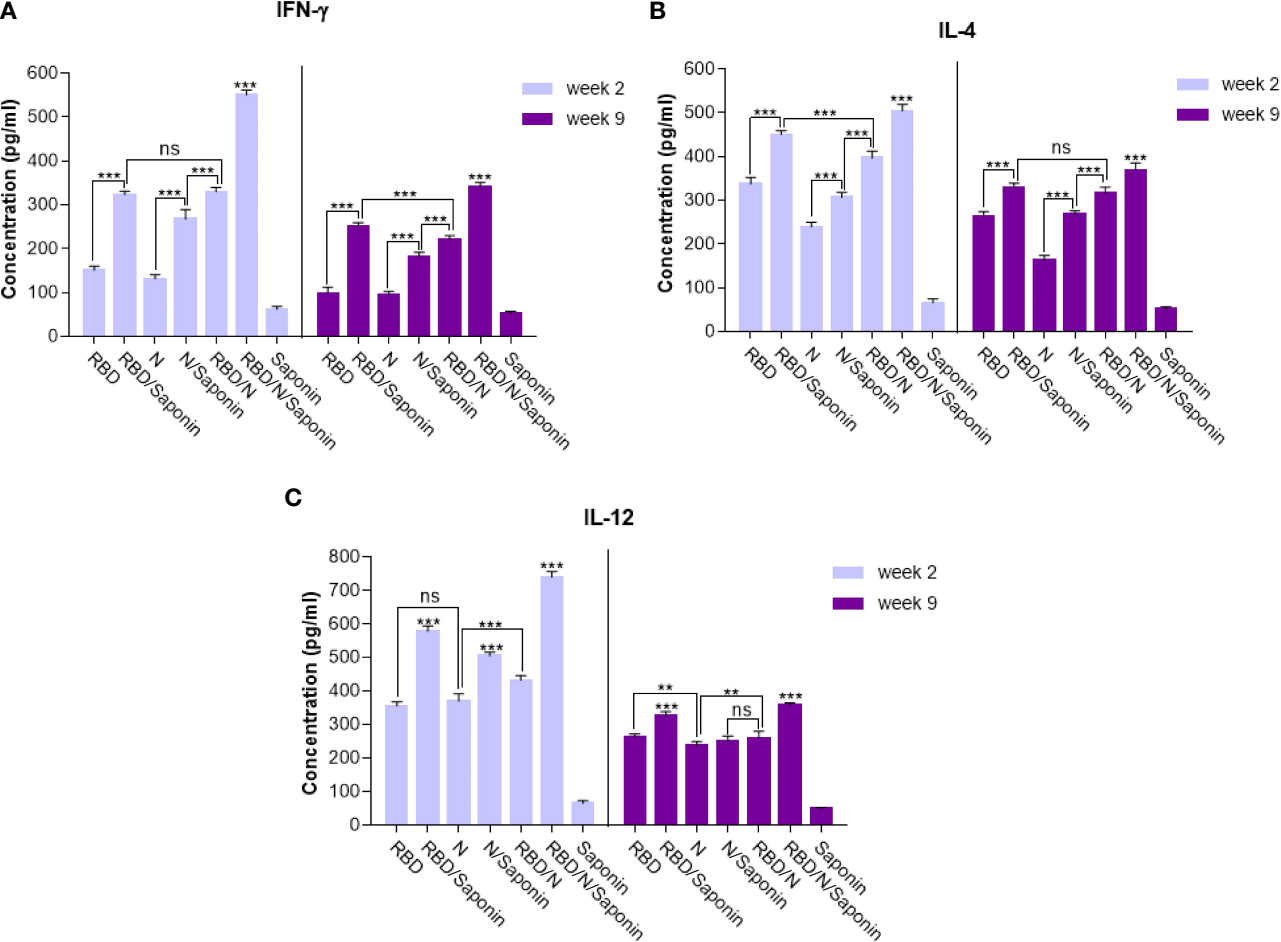
Levels of Th1/Th2 cytokine balance secreted by splenocytes in immunized mice. The supernatants of splenocytes re-stimulated with RBD/N specific epitope, collected two and nine weeks after the last immunization. ELISA method was performed to determine the level of IFN-γ **(A)**, IL-4 **(B)** and IL-12 **(C)** in splenocyte cultures. Results are representative of three independent experiments and are expressed as the mean ± SD. The RBD/N adjuvanted with Saponin induced strong levels of IFN-γ, IL-4 and IL-12 when compared to the other groups. **P < 0.01; ***P < 0.001. ns means nonstatistical difference.

The balance of protective immunity was assessed by analyzing T cell responses using the ELISA assay. Splenocytes were stimulated with synthetic epitopes that spanned the SARS-CoV-2 RBD and N proteins. Cytokine assay was conducted to monitor the balance of the cellular immunity induced by adjuvanted vaccine and to compare it with other groups. As shown in **Figure 8A**, the IFN-γ production following RBD/N/saponin vaccination was significantly (p<0.001) higher than that of all other study groups at week 2. In the same week, a higher IFN-γ level was observed in RBD/N than N/saponin (p<0.001), in N/saponin than N (p<0.001), and in RBD/saponin than RBD (p<0.001) (**Figure 8A**). Furthermore, the results showed a dramatic decrease in IFN-γ concentration at week 9 compared to week 2 among all non-control groups. Nevertheless, the IFN-γ level remained at high values for more than 2 months following the last immunization. Moreover, while the difference in IFN-γ level was negligible between RBD/N and RBD/saponin groups at week 2, we observed a significantly higher IFN-γ in RBD/saponin compared to RBD/N at week 9, highlighting the role of saponin in preserving long-term cellular immunity. The trend within other groups in week 9 was the same as in week 2.

Concerning the IL-4 level, RBD/N/saponin had the highest level of this cytokine compared to other groups at both weeks 2 and 9. After that, RBD/saponin elicited significantly higher levels of IL-4 compared to other study groups (p<0.001) at week 2. It was the same for week 9, except that RBD/saponin and RBD/N groups showed comparable levels of IL-4. Moreover, RBD/N versus N/saponin and N/saponin versus N group induced higher levels of IL-4 (both p<0.001) at both weeks 2 and 9. While a moderate decrease in IL-4 concentration within all non-control groups was observed at week 9 compared to week 2, the RBD/N/saponin showed about 8 times more IL-4 levels than control at week 9 (**Figure 8B**).

Regarding IL-12, the RBD/N/saponin group showed the highest level of this cytokine (p<0.001). Among other groups, RBD/saponin induced higher levels of IL-12 (p<0.001) at both weeks 2 and 9. Moreover, N/saponin had better performance in eliciting IL-12 (p<0.001) compared to RBD/N and N groups at both measuring weeks (**Figure 8C**). From week 2 to week 9, there was a dramatic decline in IL-12 concentration, especially for RBD/N/saponin and RBD/saponin groups. However, similar to the case of IL-4, the IL-12 concentration of RBD/N/saponin remained 8 times higher than the control.

Overall, the results indicate the efficient induction of both Th1 (according to IFN-γ and IL-12 levels) and Th2 (based on IL-4 level) responses by RBD/N/saponin formula. More importantly, The results showed a noticeable level of the cellular immune responses at longer periods, adding to the long-term protection efficacy of our developed candidate vaccine.

### RBD/N/saponin is efficient in inducing cytotoxic T cell responses as indicated by granzyme B level

**Figure 9:**
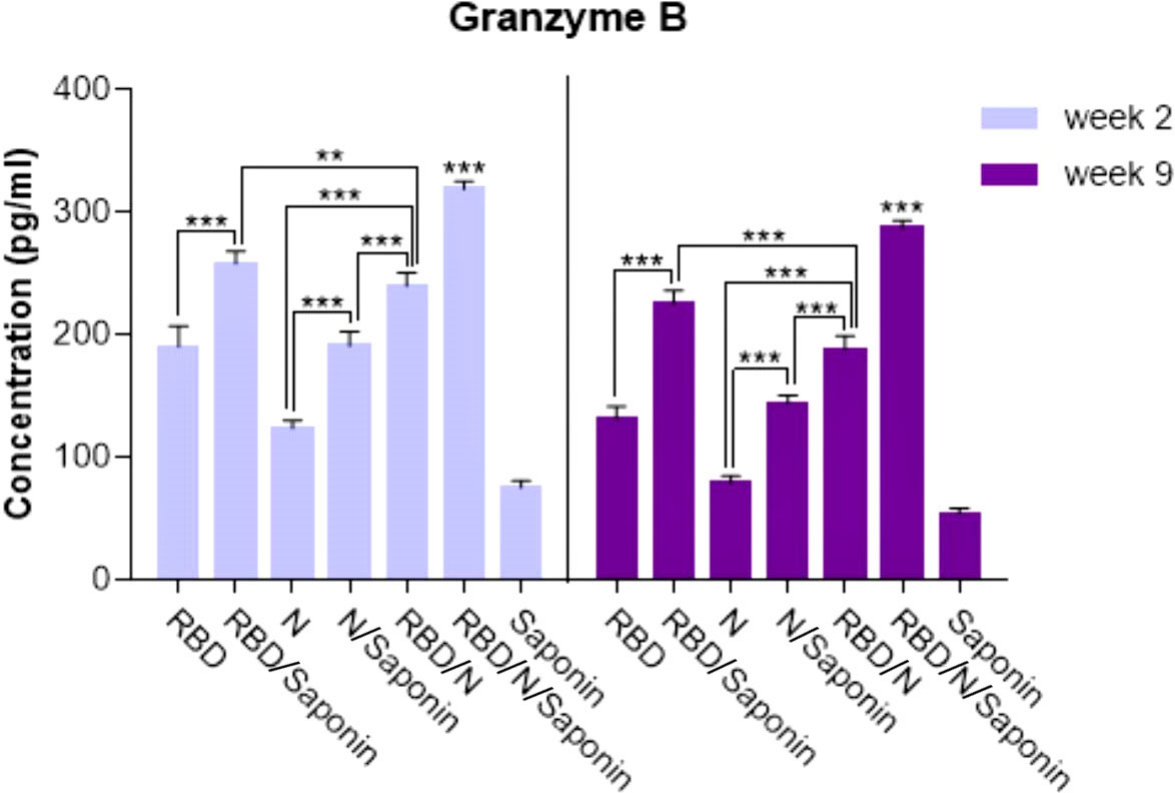
Levels of Granzyme B secreted by splenocytes in immunized mice. The supernatants of splenocytes re-stimulated with RBD/N specific epitope, collected two and nine weeks after the last immunization. ELISA method was performed to determine the level of Granzyme B in splenocyte cultures. Results are representative of three independent experiments and are expressed as the mean ± SD. The RBD/N adjuvanted with Saponin induced strong levels of Granzyme B when compared to the other groups. **P < 0.01; ***P < 0.001.

The antigen-specific cytotoxic CD8+ T cell responses elicited upon vaccination were evaluated by measuring the level of granzyme B, as the mediator of target cell death, in immunized mice 2 and 9 weeks after the last immunization. The administration of RBD/N/saponin resulted in a significantly higher granzyme B level than all study and control groups (p<0.001) at both weeks (**Figure 9**). All other study groups had a more granzyme B level than the control saponin group. Furthermore, RBD/saponin compared to RBD, N/saponin compared to N, and RBD/N compared to N/saponin showed a higher level of granzyme B (p<0.001) (**Figure 9**). Moreover, RBD/saponin induced a higher level of granzyme B compared to the RBD/N group (P<0.01 and P<0.001 for week 2 and week 9, respectively), suggesting the potent role of saponin in evoking and long-term preserving of the cytotoxic T cell responses.

## Discussion

This research investigates the immune-stimulating effect of subcutaneously injected different formulations containing recombinant SARS-CoV-2 RBD and N adjuvanted with saponin. Next, the results were compared with the immunostimulating effect of recombinant proteins alone. Based on the obtained results, the mice immunized with saponin-adjuvanted recombinant SARS-CoV-2 RBD+N harbor a high level of total IgG, IgG1, and IgG2a isotypes. Isotype switching of antibodies is influenced by cytokines, especially IFN-γ that promotes IgG2a production by Th1 and IL-4 that induces IgG1 secretion (29). We also observed that with an increase in total IgG, the neutralization capacity of the candidate vaccine increased as well. In other words, the immunized animals could produce high NAb levels just two weeks after the third immunization, suggesting the efficiency of this candidate vaccine for emergency vaccination. In addition, we studied the durability of immune responses, both humoral and cellular, for 9 weeks after the last immunization. After 9 weeks, our recombinant RBD/N/saponin candidate vaccine promisingly preserved the total IgG and IgG subclass titer at a level highly near week 2, indicating that the vaccine can achieve long-term humoral protection. This study also showed that the RBD/N/saponin candidate vaccine induced robust and long-lasting cellular immune responses. These responses were characterized by the proliferation of CD8+ and CD4+ T cells and IFN-γ production upon splenocyte stimulation with SARS-CoV-2 peptides.

Developing safe and effective vaccines to prevent SARS-CoV-2 and its variants has become the most vital priority. In this regard, producing of recombinant protein-based subunit vaccines is considered a safe approach due to the inability of these vaccines to replicate in the host (30), ensuring that the expected immune response is restricted only to the target antigen alone (31). Moreover, since there is no need to deal with a live virus during the production process of such vaccines, they are considered safe, easy-to-handle, and cost-effective vaccines (32). Some studies have employed the full-length S protein of SARS-CoV-2 as the leading antigen for this purpose. However, within the whole S protein, many non-neutralizing-epitope regions may increase the rate of antibody-dependent enhancement (ADE) of infection, regardless of non-constructive interactions. Therefore, they facilitate virus entry into the host cells that contain complement or Fc receptors and exacerbate the viral infection (33), a phenomenon also observed for some other viruses such as SARS-CoV and HIV-1 (34).

Moreover, it has been proven that the RBD fragment of SARS-CoV-2 S protein is composed of several conformational epitopes that can stimulate high titers of neutralizing antibodies and long-lasting B cell-based protective immunity (35, 36). Hence, substituting full-length S with SARS-CoV-2 RBD as the antigen not only enhances the immunogenicity of the candidate vaccine but also minimizes the risk of ADE (37, 38). However, subunit vaccines are not potent immunogens since they are not easily recognized by immune cells and are susceptible to degradation. Thus, recombinant protein vaccines often require the inclusion of adjuvants or immune stimulators to ensure long-term protective immunity (39).

As a result, many investigations have been conducted to construct COVID-19 vaccines based on RBD and by incorporating immunostimulants and adjuvants. To develop a recombinant COVID-19 subunit vaccine, RBD was fused to IgG1 Fc to enhance the candidate vaccine’s humoral and cellular immunity, maybe through facilitation of antigen presentation. Finally, it was adjuvanted with MF59, which has been shown to act as a booster in producing neutralizing antibodies and both Th1 and Th2 immune responses. This candidate vaccine could elicit high titers of neutralizing antibodies against SARS-CoV-2 (40). More interestingly, recently a self-adjuvanting RBD-based vaccine with cross-neutralization efficiency against all major known variants of SARS-CoV-2 has been developed. For this purpose, the N-terminus of RBD was chemically tagged with α-Galactosylceramide (αGalCer) and the resultant compound was assembled on the liposome surface. This conjugated candidate vaccine could induce higher level of immune responses when compared to the unconjugated version, in which the αGalCer incorporated as an adjuvant separately (41). Using synthetic Th2-skewed iNKT cell agonist (α-galactosylceramide) as the adjuvant for the RBD-Fc candidate vaccine, a highly stronger humoral immune response in the form of neutralizing antibodies was observed compared to previously reported analogous alum-adjuvanted vaccine, highlighting the importance of adjuvant in eliciting efficient immune responses (11). A candidate recombinant dimer protein COVID-19 vaccine constructed from the fusion of Interferon-α, a pan HLA-DR-binding epitope as the Th cell inducer, and Fc, for increasing the longevity of immune responses, to RBD adjuvanted with alum, showed promising immunization in the phase-I clinical trial. Sufficient immunogenicity and robust immune responses were reported as the trial’s outcome (42).

Beside S, N protein as a structural compartment of SARS-CoV-2 has shown potent immunogenic signs (43, 44). N is more conserved and stable than S. Hence, recombinant vaccines that incorporate N as the main or supplementary antigen may be more trustworthy to be efficient in providing immunity over time when new variants of the virus emerge (45, 46). Furthermore, the N protein of SARS-CoV-2 harnesses the densest epitopic region targeted by memory CD8+ T cells. Meanwhile, the share of S is very low, highlighting the key role of N in inducing long-lasting immunity (47).

Moreover, the serological evaluation of virus-specific T cells and their impact on neutralizing antibodies in convalescent COVID-19 patients showed a tight correlation between the level of neutralizing antibodies and the number of T cells specific to N protein. These results suggest the involvement of both T and B cells in immunity against SARS-CoV-2 (48). The accumulation of N-specific T cells also has been reported in the lung of mice immunized with recombinant adenovirus type‐5 expressing SARS‐CoV‐2 N protein (49). Here, we incorporated N into the recipe of our candidate vaccine to simultaneously harness neutralizing antibody responses generated against both RBD and N antigens and T cell-mediated immunity, thereby promoting the longevity and quality of protection against SARS-CoV-2 infection.

A mixture of antigens composed of SARS-CoV-2 RBD and N proteins co-expressed in *Nicotiana benthamiana* plant recently indicated potential in inducing high titer of antibodies. To our knowledge, no study has been conducted on potential cellular immunity mediated by that candidate vaccine. However, the authors hypothesized that while no neutralizing activity was observed when N protein was administered separately, its presence adds long-lasting and more efficient protective properties to the recombinant vaccine rather than RBD alone (50). In the present study, we employed a robust bacterial expression system to develop a newly formulated recombinant RBD/N vaccine. We further equipped it with saponin as an immunopotentiator adjuvant. The purified proteins were detected by Western blot using monoclonal antibodies containing different conformational and linear epitopes in RBD (51). The results suggest that the recombinant proteins maintain intact spatial conformation and authentic antigenicity. Also, our findings showed that the combination of RBD and N proteins with saponin significantly promoted specific neutralization antibodies, eliciting robust specific lymphoproliferative and T-cell responses. In addition, a remarkable increase in multifunctional CD4+ and CD8+ T cells was observed following vaccination with adjuvanted RBD/N vaccine. The results suggest that our formulation is able to elicit a mixed Th1/Th2 balanced immune response.

In a recent study, the bacterial RBD was structurally characterized and compared with the RBD expressed by HEK293 cells. The results showed that the secondary and tertiary structure of prokaryotic proteins are highly conserved and could strongly bind ACE2 (21). The findings suggest that the absence of glycosylation could partially affect ACE2 binding *in vitro* (52). In another study and in parallel with HEK-293 and insect cells, the *E. coli*-expressed RBD characterization showed the ability of the bacterial expression system in producing high-quality and antibody-recognizable proteins (53).

Regarding the virus-host interaction, glycans on the surface of viral proteins can play contradictory dual roles. On one hand, they may facilitate the antibody recognition and act like epitopes. On the other hand, they may shield the epitopic regions on viral proteins and thereby hamper efficient antibody production (54–56). In this regard, lack of glycosylation in *E. coli*-expressed proteins can be considered as a drawback of this expression system for the development of recombinant vaccines. However, our data clarify that the production of antibodies specific to the SARS-CoV-2 RBD and N fragments is at a level that seemingly still justifies the use of bacterial expression system. A detailed comparative study can better elucidate if, and to what extent, the type of expression system may affect the level of antibody production.

To present recombinant protein vaccines to CD8+ T cells by dendritic cells and stimulate sufficient and effective immune responses, they need to be supplemented with immunopotentiator adjuvants. Adjuvants developed based on saponins lead immune responses toward Th1 and CTL responses mediated by augmentation in release of IFN-γ by dendritic cells, thereby resulting in efficient humoral and cellular immune responses (20). In support, the saponin-adjuvanted recombinant RBD/N vaccine reported in this study showed robust immunogenicity and efficacy in mice. Hence, it is a potent COVID-19 vaccine candidate and worth further development. It has been shown that saponin can motivate cellular immunity and stimulate antibody induction (57). The adjuvant also could increase the duration of the immune response and allow a major dose reduction for the vaccine antigen (58). The present study showed that saponin had a significant adjuvant effect and induced a strong humoral and cellular immune response against the RBD. During the 9-week follow-up, the titer of virus-NAb did not significantly decrease. These results provide immunogenicity data for a vaccine using RBD expressed by a bacterial host as the immunogen.

## Conclusion

Subunit vaccines, constructed based on recombinant antigen proteins with promising immunogenicity, can stimulate host immunity. Our recombinant bacterially-expressed RBD/N vaccine adjuvanted with saponin demonstrated immunogenicity in the BALB-c mouse model. If reproduced in humans in ongoing studies, the vaccine could potentially be administered in humans for protection against COVID-19. Regarding the ease of production, robust and efficient antiviral responses, and durability of immune responses, the RBD/N/saponin candidate vaccine is highly worth investigating in clinical trials. However, as the candidate vaccine investigated here is a newly formulated SARS-CoV-2 recombinant vaccine produced in *E. coli*, it will be beneficial to compare the results of this study with a similar approach using a distinct expression system and balance the advantages and disadvantages of both systems and then decide on further course of action.

## Data availability statement

The original contributions presented in the study are included in the article/supplementary material. Further inquiries can be directed to the corresponding author.

## Ethics statement

The animal study was reviewed and approved by the Iranian Biotechnology Development Council.

## Author contributions

AG and AH conceived and designed the study. PRA, HZ, HA EA, and SB performed experiments. AG and SM wrote the manuscript. AG, PRA, SM, and AH assisted in experimental design and preparation of the manuscript. AG and SM supervised the study. All authors contributed to the article and approved the submitted version.

## Funding

This work was supported by Iranian Biotechnology Development Council.

## Conflict of interest

Authors HZ, AH, EA and SM were employed by TRS Biotech Company.

The remaining authors declare that the research was conducted in the absence of any commercial or financial relationships that could be construed as a potential conflict of interest.

## Publisher’s note

All claims expressed in this article are solely those of the authors and do not necessarily represent those of their affiliated organizations, or those of the publisher, the editors and the reviewers. Any product that may be evaluated in this article, or claim that may be made by its manufacturer, is not guaranteed or endorsed by the publisher.

## References

[1] Yang Y Xiao Z Ye K He X Sun B Qin Z. SARS-CoV-2: characteristics and current advances in research. Virol J (2020) 17(1):1–17. doi: 10.1186/s12985-020-01369-z 32727485 PMC7387805

[2] Thomas S Abraham A Callaghan PJ Rappuoli R. Challenges for vaccinologists in the first half of the twenty-first century. Vaccine Des. Springer (2022) p:3–25. doi: 10.1007/978-1-0716-1884-4_1 34914040

[3] Alam A Rampes S Ma D. The impact of the COVID-19 pandemic on research. Transl Perioper Pain Med (2021) 8(1):312–4. doi: 10.1097/PR9.0000000000000891

[4] Zhu Y Yu D Yan H Chong H He Y. Design of potent membrane fusion inhibitors against SARS-CoV-2, an emerging coronavirus with high fusogenic activity. J Virol (2020) 94(14):e00635–20. doi: 10.1128/JVI.00635-20 PMC7343218 32376627

[5] Al-Jighefee HT Najjar H Ahmed MN Qush A Awwad S Kamareddine L. COVID-19 vaccine platforms: Challenges and safety contemplations. Vaccines (2021) 9(10):1196. doi: 10.3390/vaccines9101196 34696306 PMC8537163

[6] Chaudhary JK Yadav R Chaudhary PK Maurya A Kant N Rugaie OA. Insights into COVID-19 vaccine development based on immunogenic structural proteins of SARS-CoV-2, host immune responses, and herd immunity. Cells (2021) 10(11):2949. doi: 10.3390/cells10112949 34831172 PMC8616290

[7] Wagner A Guzek A Ruff J Jasinska J Scheikl U Zwazl I. Neutralising SARS-CoV-2 RBD-specific antibodies persist for at least six months independently of symptoms in adults. Commun Med (2021) 1(1):1–11. doi: 10.1038/s43856-021-00012-4 35602189 PMC9037317

[8] Muecksch F Wang Z Cho A Gaebler C Tanfous TB DaSilva J. Increased potency and breadth of SARS-CoV-2 neutralizing antibodies after a third mRNA vaccine dose. bioRxiv (2022). doi: 10.1101/2022.02.14.480394

[9] Gong Y Qin S Dai L Tian Z. The glycosylation in SARS-CoV-2 and its receptor ACE2. Signal transduct. target. Ther (2021) 6(1):1–24. doi: 10.1038/s41392-021-00809-8 34782609 PMC8591162

[10] Pérez-Rodríguez S de la Caridad Rodríguez-González M Ochoa-Azze R Climent-Ruiz Y González-Delgado CA Paredes-Moreno B. A randomized, double-blind phase I clinical trial of two recombinant dimeric RBD COVID-19 vaccine candidates: safety, reactogenicity and immunogenicity. Vaccine (2022) 18;40(13):2068–75. doi: 10.1016/j.vaccine.2022.02.029 PMC8823954 35164986

[11] Wang X-F Zhang M-J He N Wang Y-C Yan C Chen X-Z. Potent neutralizing antibodies elicited by RBD-Fc-Based COVID-19 vaccine candidate adjuvanted by the Th2-skewing iNKT cell agonist. J med. Chem (2021) 64(15):11554–69. doi: 10.1021/acs.jmedchem.1c00881 34279930

[12] Jeong H Choi Y-M Seo H Kim B-J. A novel DNA vaccine against SARS-CoV-2 encoding a chimeric protein of its receptor-binding domain (RBD) fused to the amino-terminal region of hepatitis b virus preS1 with a W4P mutation. Front Immunol (2021) 12:482. doi: 10.3389/fimmu.2021.637654 PMC7959807 33732258

[13] Yang S Li Y Dai L Wang J He P Li C. Safety and immunogenicity of a recombinant tandem-repeat dimeric RBD-based protein subunit vaccine (ZF2001) against COVID-19 in adults: two randomised, double-blind, placebo-controlled, phase 1 and 2 trials. Lancet Infect Dis (2021) 21(8):1107–19. doi: 10.1016/S1473-3099(21)00127-4 PMC7990482 33773111

[14] Sun W He L Zhang H Tian X Bai Z Sun L. The self-assembled nanoparticle-based trimeric RBD mRNA vaccine elicits robust and durable protective immunity against SARS-CoV-2 in mice. Signal transduct. target. Ther (2021) 6(1):1–11. doi: 10.1038/s41392-021-00750-w 34504054 PMC8426336

[15] Yang J Wang W Chen Z Lu S Yang F Bi Z. A vaccine targeting the RBD of the s protein of SARS-CoV-2 induces protective immunity. Nature (2020) 586(7830):572–7. doi: 10.1038/s41586-020-2599-8 32726802

[16] Routhu NK Cheedarla N Bollimpelli VS Gangadhara S Edara VV Lai L. SARS-CoV-2 RBD trimer protein adjuvanted with alum-3M-052 protects from SARS-CoV-2 infection and immune pathology in the lung. Nat Commun (2021) 12(1):1–15. doi: 10.1038/s41467-021-23942-y 34117252 PMC8196016

[17] Wang J Yin X-G Wen Y Lu J Zhang R-Y Zhou S-H. MPLA-Adjuvanted liposomes encapsulating s-trimer or RBD or S1, but not s-ECD, elicit robust neutralization against SARS-CoV-2 and variants of concern. J med. Chem (2022) 65(4):3563–74. doi: 10.1021/acs.jmedchem.1c02025 35108485

[18] Dangi T Class J Palacio N Richner JM MacMaster PP. Combining spike-and nucleocapsid-based vaccines improves distal control of SARS-CoV-2. Cell Rep (2021) 36(10):109664. doi: 10.1016/j.celrep.2021.109664 34450033 PMC8367759

[19] Fu Y Chen F Cui L Zhao Y Zhang H Fu S. Immunological analysis of people in northeast China after SARS-CoV-2 inactivated vaccine injection. Vaccines (2021) 9(9):1028. doi: 10.3390/vaccines9091028 34579267 PMC8473348

[20] Sharma R Palanisamy A Dhama K Mal G Singh B Singh KP. Exploring the possible use of saponin adjuvants in COVID-19 vaccine. Hum Vaccines Immunother. (2020) 16(12):2944–53. doi: 10.1080/21645515.2020.1833579 PMC7738204 33295829

[21] He Y Qi J Xiao L Shen L Yu W Hu T. Purification and characterization of the receptor-binding domain of SARS-CoV-2 spike protein from escherichia coli. Eng Life Sci (2021) 21(6):453–60. doi: 10.1002/elsc.202000106 PMC8182281 34140855

[22] Sabbaghi A Malek M Abdolahi S Miri SM Alizadeh L Samadi M. A formulated poly (I:C)/CCL21 as an effective mucosal adjuvant for gamma-irradiated influenza vaccine. Virol J (2021) 18(1):201. doi: 10.1186/s12985-021-01672-3 34627297 PMC8501930

[23] Sabbaghi A Zargar M Zolfaghari MR Motamedi-Sedeh F Ghaemi A. Protective cellular and mucosal immune responses following nasal administration of a whole gamma-irradiated influenza a (subtype H1N1) vaccine adjuvanted with interleukin-28B in a mouse model. Arch Virol (2021) 166(2):545–57. doi: 10.1007/s00705-020-04900-3 PMC7787640 33409549

[24] Quiros-Fernandez I Poorebrahim M Fakhr E Cid-Arregui A. Immunogenic T cell epitopes of SARS-CoV-2 are recognized by circulating memory and naïve CD8 T cells of unexposed individuals. EBioMedicine (2021) 72:103610. doi: 10.1016/j.ebiom.2021.103610 34627082 PMC8493415

[25] Harris PE Brasel T Massey C Herst C Burkholz S Lloyd P. A synthetic peptide CTL vaccine targeting nucleocapsid confers protection from SARS-CoV-2 challenge in rhesus macaques. Vaccines (2021) 9(5):520. doi: 10.3390/vaccines9050520 34070152 PMC8158516

[26] Rammensee H-G Gouttefangeas C Heidu S Klein R Preuß B Walz JS. Designing a sars-cov-2 t-cell-inducing vaccine for high-risk patient groups. Vaccines (2021) 9(5):428. doi: 10.3390/vaccines9050428 33923363 PMC8146137

[27] Dakal TC. Antigenic sites in SARS-CoV-2 spike RBD show molecular similarity with pathogenic antigenic determinants and harbors peptides for vaccine development. Immunobiology (2021) 226(5):152091. doi: 10.1016/j.imbio.2021.152091 34303920 PMC8297981

[28] Baghban Rahimi S Mohebbi A Vakilzadeh G Biglari P Razeghi Jahromi S Mohebi SR. Enhancement of therapeutic DNA vaccine potency by melatonin through inhibiting VEGF expression and induction of antitumor immunity mediated by CD8+ T cells. Arch Virol (2018) 163(3):587–97. doi: 10.1007/s00705-017-3647-z 29149434

[29] Stavnezer J. Immunoglobulin class switching. Curr Opin Immunol (1996) 8(2):199–205. doi: 10.1016/S0952-7915(96)80058-6 8725943

[30] Lee N-H Lee J-A Park S-Y Song C-S Choi I-S Lee J-B. A review of vaccine development and research for industry animals in Korea. Clin Exp Vaccine Res (2012) 1(1):18–34. doi: 10.7774/cevr.2012.1.1.18 23596575 PMC3623508

[31] Wang M Jiang S Wang Y. Recent advances in the production of recombinant subunit vaccines in pichia pastoris. Bioengineered (2016) 7(3):155–65. doi: 10.1080/21655979.2016.1191707 PMC4927204 27246656

[32] Bellini C Horváti K. Recent advances in the development of protein-and peptide-based subunit vaccines against tuberculosis. Cells (2020) 9(12):2673. doi: 10.3390/cells9122673 PMC7765234 33333744

[33] Wang Z Deng T Zhang Y Niu W Nie Q Yang S. ACE2 can act as the secondary receptor in the FcγR-dependent ADE of SARS-CoV-2 infection. Iscience (2022) 25(1):103720. doi: 10.1016/j.isci.2021.103720 35005526 PMC8719361

[34] Taylor A Foo SS Bruzzone R Vu Dinh L King NJ Mahalingam S. Fc receptors in antibody-dependent enhancement of viral infections. Immunol Rev (2015) 268(1):340–64. doi: 10.1111/imr.12367 PMC7165974 26497532

[35] Abayasingam A Balachandran H Agapiou D Hammoud M Rodrigo C Keoshkerian E. Long-term persistence of RBD+ memory b cells encoding neutralizing antibodies in SARS-CoV-2 infection. Cell Rep Med (2021) 2(4):100228. doi: 10.1016/j.xcrm.2021.100228 33748788 PMC7955929

[36] Li Y M-l Ma Lei Q Wang F Hong W D-y L. Linear epitope landscape of the SARS-CoV-2 spike protein constructed from 1,051 COVID-19 patients. Cell Rep (2021) 34(13):108915. doi: 10.1016/j.celrep.2021.108915 33761319 PMC7953450

[37] Bayarri-Olmos R Idorn M Rosbjerg A Pérez-Alós L Hansen CB Johnsen LB. SARS-CoV-2 neutralizing antibody responses towards full-length spike protein and the receptor-binding domain. J Immunol (2021) 207(3):878–87. doi: 10.4049/jimmunol.2100272 34301847

[38] Lee WS Wheatley AK Kent SJ DeKosky BJ. Antibody-dependent enhancement and SARS-CoV-2 vaccines and therapies. Nat Microbiol (2020) 5(10):1185–91. doi: 10.1038/s41564-020-00789-5 PMC12103240 32908214

[39] Skwarczynski M Toth I. Peptide-based synthetic vaccines. Chem Sci (2016) 7(2):842–54. doi: 10.1039/C5SC03892H PMC5529997 28791117

[40] Liu X Drelich A Li W Chen C Sun Z Shi M. Enhanced elicitation of potent neutralizing antibodies by the SARS-CoV-2 spike receptor binding domain fc fusion protein in mice. Vaccine (2020) 38(46):7205–12. doi: 10.1016/j.vaccine.2020.09.058 PMC7508516 33010978

[41] Wang J Wen Y Zhou S-H Zhang H-W Peng X-Q Zhang R-Y. Self-adjuvanting lipoprotein conjugate αGalCer-RBD induces potent immunity against SARS-CoV-2 and its variants of concern. J med. Chem (2022) 65(3):2558–70. doi: 10.1021/acs.jmedchem.1c02000 35073081

[42] Zhang J Hu Z He J Liao Y Li Y Pei R. Safety and immunogenicity of a recombinant interferon-armed RBD dimer vaccine (V-01) for COVID-19 in healthy adults: a randomized, double-blind, placebo-controlled, phase I trial. Emerg. Microbes infect. (2021) 10(1):1589. doi: 10.1080/22221751.2021.1951126 34197281 PMC8366678

[43] Dobaño C Santano R Jiménez A Vidal M Chi J Melero NR. Immunogenicity and crossreactivity of antibodies to the nucleocapsid protein of SARS-CoV-2: utility and limitations in seroprevalence and immunity studies. Trans Res (2021) 232:60–74. doi: 10.1016/j.trsl.2021.02.006 PMC7879156 33582244

[44] Ahlén G Frelin L Nikouyan N Weber F Höglund U Larsson O. The SARS-CoV-2 n protein is a good component in a vaccine. J Virol (2020) 94(18):e01279–20. doi: 10.1128/JVI.01279-20 PMC7459553 32661140

[45] Oliveira SC de Magalhães MT Homan EJ. Immunoinformatic analysis of SARS-CoV-2 nucleocapsid protein and identification of COVID-19 vaccine targets. Front Immunol (2020) 2758. doi: 10.3389/fimmu.2020.587615 PMC7655779 33193414

[46] Grifoni A Sidney J Zhang Y Scheuermann RH Peters B Sette A. A sequence homology and bioinformatic approach can predict candidate targets for immune responses to SARS-CoV-2. Cell Host Microbe (2020) 27(4):671–680.e2. doi: 10.1016/j.chom.2020.03.002 32183941 PMC7142693

[47] Ferretti AP Kula T Wang Y Nguyen DM Weinheimer A Dunlap GS. Unbiased screens show CD8+ T cells of COVID-19 patients recognize shared epitopes in SARS-CoV-2 that largely reside outside the spike protein. Immunity (2020) 53(5):1095–1107.e3. doi: 10.1016/j.immuni.2020.10.006 33128877 PMC7574860

[48] Ni L Ye F Cheng M-L Feng Y Deng Y-Q Zhao H. Detection of SARS-CoV-2-specific humoral and cellular immunity in COVID-19 convalescent individuals. Immunity (2020) 52(6):971–977.e3. doi: 10.1016/j.immuni.2020.04.023 32413330 PMC7196424

[49] He J Huang JR Zhang YL Zhang J. SARS-CoV-2 nucleocapsid protein intranasal inoculation induces local and systemic T cell responses in mice. J Med Virol (2021) 93(4):1923–5. doi: 10.1002/jmv.26769 33386773

[50] Mamedov T Yuksel D Ilgın M Gürbüzaslan I Gulec B Mammadova G. Production and characterization of nucleocapsid and RBD cocktail antigens of SARS-CoV-2 in nicotiana benthamiana plant as a vaccine candidate against COVID-19. Vaccines (2021) 9(11):1337. doi: 10.3390/vaccines9111337 34835268 PMC8621474

[51] He Y Lu H Siddiqui P Zhou Y Jiang S. Receptor-binding domain of severe acute respiratory syndrome coronavirus spike protein contains multiple conformation-dependent epitopes that induce highly potent neutralizing antibodies. J Immunol (2005) 174(8):4908–15. doi: 10.4049/jimmunol.174.8.4908 15814718

[52] Azad T Singaravelu R Taha Z Jamieson TR Boulton S Crupi MJ. Nanoluciferase complementation-based bioreporter reveals the importance of n-linked glycosylation of SARS-CoV-2 s for viral entry. Mol Ther (2021) 29(6):1984–2000. doi: 10.1016/j.ymthe.2021.02.007 33578036 PMC7872859

[53] Maffei M Montemiglio LC Vitagliano G Fedele L Sellathurai S Bucci F. The nuts and bolts of SARS-CoV-2 spike receptor-binding domain heterologous expression. Biomolecules (2021) 11(12):1812. doi: 10.3390/biom11121812 34944456 PMC8699011

[54] Zhao X Chen H Wang H. Glycans of SARS-CoV-2 spike protein in virus infection and antibody production. Front Mol Biosci (2021) 8:629873. doi: 10.3389/fmolb.2021.629873 33928117 PMC8076860

[55] Casalino L Gaieb Z Goldsmith JA Hjorth CK Dommer AC Harbison AM. Beyond shielding: the roles of glycans in the SARS-CoV-2 spike protein. ACS Cent Sci (2020) 6(10):1722–34. doi: 10.1021/acscentsci.0c01056 PMC7523240 33140034

[56] Lemmin T Kalbermatter D Harder D Plattet P Fotiadis D. Structures and dynamics of the novel S1/S2 protease cleavage site loop of the SARS-CoV-2 spike glycoprotein. J Struct biol.: X (2020) 4:100038. doi: 10.1016/j.yjsbx.2020.100038 33043289 PMC7534663

[57] Cibulski S Rivera-patron M Suárez N Pirez M Rossi S Carolina A. Leaf saponins of quillaja brasiliensis enhance long-term specific immune responses and promote dose-sparing effect in BVDV experimental vaccines. Vaccine [Internet] (2017) 36(1):55–65. doi: 10.1016/j.vaccine.2017.11.030 29174676

[58] Cibulski SP Mourglia-Ettlin G Teixeira TF Quirici L Roehe PM Ferreira F. Novel ISCOMs from quillaja brasiliensis saponins induce mucosal and systemic antibody production, T-cell responses and improved antigen uptake. Vaccine (2016) 34(9):1162–71. doi: 10.1016/j.vaccine.2016.01.029 26826546

